# Mapping drivers of sexual and reproductive health injustices among adolescent girls in Kenya, Ghana, and Zambia: a scoping review

**DOI:** 10.1186/s12889-026-28865-1

**Published:** 2026-08-11

**Authors:** Dona Anyona, Rieke van der Graaf, Fleur Kootstra, Josephat Nyagero, Julie Fraser, Johannes J. M. van Delden

**Affiliations:** 1 Department of Bioethics and Health Humanities, Julius Center for Health Sciences and Primary Care, University Medical Center Utrecht, Utrecht University, Utrecht, The Netherlands; 2 Amref International University, Nairobi, Kenya; 3 Utrecht University School of Law, Institute of Human Rights, Utrecht, The Netherlands

**Keywords:** Reproductive health, Sexual health, Reproductive well-being, Adolescents, Injustices

## Abstract

**Background:**

Violation of adolescent girls’ sexual reproductive health rights (SRHR) leads to injustices that impact their well-being with potentially fatal and life-long consequences. These injustices often arise due to early marriage, and in 2022, approximately 12 million girls were married before the age of 18, globally. Adolescent girls’ SRHR is especially problematic in Africa, with the highest levels being from West and Central Africa (40%), followed by East and Southern Africa (32%). To address the problem of adolescent girls’ SRHR violations, interventions have included formulating laws and policies to protect the rights and welfare of girls in national legislation, as well as in regional and international human rights instruments. Despite these efforts, the literature that explicitly focuses on SRHR lacks mapping related to drivers of adolescent girls’ SRH injustices to inform programming and policies. Therefore, a scoping review was necessary to map and understand what drives these injustices of adolescent girls’ SRHR in Africa.

**Methods:**

We conducted a scoping review of papers using a SRHR lens to identify the drivers of adolescent girls’ SRHR injustices in Kenya, Ghana, and Zambia, drawing on multidisciplinary papers. We utilised the Web of Science, PubMed, HeinOnline, JSTOR, Scopus, ScienceDirect, and HINARI databases. Drivers of adolescent girls’ SRHR injustices were extracted from the papers and thematically categorised.

**Results:**

We identified 18 papers on adolescent girls’ SRHR from Kenya, Ghana, and Zambia. The main drivers therein included poverty, culture and gender norms, discrimination based on disability, low education level, peer pressure, and limited access to SRH services, such as financial and geographic barriers. The main issue that cut across all these drivers was adolescent pregnancy.

**Conclusion:**

From the papers, using an SRHR frame, poverty is one of the main drivers that stands out and interconnects with others, including lack of education, lack of access to SRH services, and culture. All of the identified drivers were related to adolescent pregnancy. While adolescent pregnancy in itself may not be a human rights violation, it may relate to violations such as lack of access to services, lack of education, and early child marriage. The outcomes of the review will inform policy interventions to protect adolescent girls’ SRHR.

**Supplementary Information:**

The online version contains supplementary material available at https://doi.org/10.1186/s12889-026-28865-1.

## Introduction

Adolescents, who comprise 1.2 billion people according to the WHO’s Global Accelerated Action for Health of Adolescents (2017) report, are at a critical stage in their lifelong health. Therefore, interventions during this period have a lasting impact on adulthood, including educational and economic outcomes. It is imperative to explore the injustices that may hinder their development, especially in low- and middle-income countries. Adolescent girls’ sexual reproductive health rights (ASRHR) injustices arise from structural and systemic issues that prevent adolescents from accessing sexual reproductive services and rights that concern them [1, 2].

Sexual reproductive health violations are those acts that occur when state or non-state actors fail to act on their obligations to protect or promote adolescent girls’ human rights [1, 2]. SRHR is a state of physical, emotional, mental, and social well-being where individuals have a right to make decisions governing their bodies and to access services that support that right [2]. The elements of this definition adopt a holistic approach to tackling neglected issues, such as adolescent sexuality. Violations in this context are defined from a sexual gender based violence perspective that includes harmful acts perpetrated against a person’s will based on socially ascribed differences. They are often due to deeply ingrained beliefs and societal values pertaining to women’s sexuality.

Adolescent sexual reproductive health injustice is used in the context of systematic exclusion from the full enjoyment of their human rights due to discrimination, inequality, or failure by duty bearers to protect and fulfil those rights. The violation of adolescent girls’ SRHR, therefore, leads to injustices that impact their well-being with potentially fatal or life-long consequences. These injustices result from various issues such as limited access to comprehensive sex education [1, 2], age-related barriers to access contraceptives [3], harmful cultural attitudes [4], gender-based discrimination and violence [5], as well as female genital mutilation [6], which are also human rights violations [7]. Child marriage is the most common form of children’s rights violation worldwide, despite most countries, including in sub-Saharan Africa (SSA), setting the legal age for marriage at 18 years [8].

Papers estimate that each year, approximately 12 million girls are married before the age of 18, globally, with the highest levels being in West and Central Africa, where nearly 4 in 10 girls were married before the age of 18, followed by East and Southern Africa, where the level of child marriage is over 3 in 10 girls [8]. Such practices lead to the further violation of these girls’ rights, including the supreme right to life in cases where girls die due to complications of early/multiple pregnancies. Papers report that the adolescent maternal mortality rate in SSA is as high as 260 per 100,000 live births, more than the 190 per 100,000 live births for adults above the age of 19 recorded in 2014 [9].

Diverse human rights law frameworks protect adolescent girls’ sexual and reproductive health. Adolescent girls’ SRHR are specifically protected by the UN International Covenant on Economic, Social, and Cultural Rights (ICESCR) [10], the Convention on the Elimination of all Forms of Discrimination Against Women (CEDAW) [5], and the Convention on the Rights of the Child (CRC). Article 24 of the CRC protects the health of children, categorised as anyone under 18 years, and Article 34 protects them from any form of sexual exploitation [7]. According to Articles 2 and 12 of the ICESCR, states must guarantee the enjoyment of the highest attainable standard of physical and mental health, using the maximum available resources [11].

At the continental level, African regional human rights treaties and protocols also remain relevant in protecting similar rights, emphasising SRHR, especially for children. For example, Articles 12 and 21 of the African Charter on the Rights and Welfare of the Child [11] and Article 13 of the Maputo Protocol [12] relate to the promotion of the right to health and protection from harmful social and cultural practices relating to SRHR and protection from exploitation of children. Furthermore, the International Conference on Population and Development (ICPD) [13] and Sustainable Development Goal (SDG) 3 [14] continue to initiate global SRHR conversations and commitments, which have been articulated in Goal 3.7 that clarifies the importance of access to SRHR [15].

These frameworks led to a plethora of national laws and policies, enabling states to meet targets and achieve ASRHR. Despite this, injustices persist. Many low- and middle-income countries, particularly in Africa, have not yet made significant progress in delaying marriage and childbearing, reducing unwanted pregnancies, or reducing socio-economic, health, and gender inequalities that could decrease girls’ risks of adverse adolescent sexual and reproductive health [16]. For example, a 2020 report by the United Nations Population Fund (UNFPA), WHO, and Federal Centre for Health Education confirms that adolescent pregnancy is a major injustice for girls aged 15 to 19 in low-and middle-income countries [17]. Another report by the UNAIDS also examines unequal gender norms, gender-based violence, early teen girls’ pregnancies, and lack of sufficient sexual and reproductive care as injustices [18].

This situation highlights the need to take further action in addressing persistent problems by uncovering the complex dynamics and root causes underlying these SRHR injustices. Although a few of these dynamics, such as specific cultural norms/practices and lack of education, have been identified by organisations, including the United Nations Development Programme (UNDP) [19] and WHO [1, 2] as significant causes of early pregnancy, their persistence implies that we need to identify which drivers emerge from literature that explicitly focuses on SRHR. UNDP [11] and WHO [18] recommendations highlight the need to understand the various causal factors to determine why legal frameworks are not implemented. The UNDP has reported that addressing the root causes of ASRH issues requires a context-specific understanding of the underlying causal factors [5, 20], and the UNFPA has raised similar concerns [21]. The UNFPA further [22] considers SRHR challenges an ongoing issue in Kenya, while UNICEF [23] and Chilambe et al. [24] confirm the multiple SRH violations in Ghana and Zambia.

The selection of Kenya, Ghana, and Zambia for this scoping review considers all three countries, which are Commonwealth countries operating within legal systems influenced by English common law. The shared legal and institutional heritage provides a unique basis for comparative analysis, particularly in ASRHR, where legal frameworks, policy development, and programmatic interventions often intersect. These countries also demonstrate both similarities and contrasts in socio-cultural norms and policy implementation environments, making them ideal for identifying cross-cultural drivers of adolescent girls’ SRHR and context-specific nuances.

Focusing on these three countries captures the diversity of experiences within SSA, which offers relevance to the review’s findings. Kenya, Ghana, and Zambia are recognised for their engagement with regional and international commitments to adolescent health and gender equality, such as the Convention on the Elimination of All Forms of Discrimination Against Women, the Convention on the Rights of the Child, the African Union’s Agenda 2063, the Maputo Plan of Action, and the Sustainable Development Goals. Existing ASRHR literature may explore injustices independently without framing the rights discourse, thereby affecting the understanding of interconnectedness between injustices and the underlying drivers.

To address this gap, this scoping review searched papers that identified and framed themselves as addressing SRHR. We did not search for specific examples such as FGM or Intimate Partner Violence (IPV), because we wanted to study in general what the issues were. We did not attempt to explore all papers on SRHR injustices without a specific SRHR discourse framing. Instead, we explored the conceptualization of adolescent SRHR injustices and discussions within literature that specifically focuses on SRHR.

### Research question

What are the drivers of injustices of adolescent girls’ sexual and reproductive health rights in Kenya, Zambia, and Ghana?

## Methods

### Scoping review

We conducted a scoping review of papers from multidisciplinary areas regarding the drivers of adolescent girls’ SRHR injustices in Kenya, Ghana, and Zambia. Due to colonisation, these countries share legal and policy frameworks rooted in English Common Law, which continue to influence their current sexual and reproductive health legislation and enforcement mechanisms. The selected countries represent the West, East, and South of Africa. The UNFPA considers SRHR challenges an ongoing issue in Kenya, while UNICEF and Chilambe et al. [25] confirm the multiple SRH violations in Ghana and Zambia. The review was also limited to these countries because they represent some of the SSA countries with significant ASRHR injustices [25]. UNFPA acknowledges that many SSA struggle with ASRH injustices despite the diverse regulations, [19] including Kenya and Ghana, as part of low and middle-income countries, and Zambia, classified as a lower-income country, with sexual and reproductive health injustices among different age groups due to associated environmental, social, and economic challenges. These countries emerged as preferable case studies because they are also leading members of the Countdown to 2030, whose primary goal is advocating for adolescents, women, and children’s health [26] and SRHR having flagged the countries’ concluding observations of the CEDAW committee, which noted the need for comprehensive sexuality education for adolescents and protection of girls from early marriages [27–29]. Further, Kenya, Ghana, and Zambia are recognised for their engagement with regional and international commitments to adolescent health and gender equality, such as the CEDAW, CRC, the African Union’s Agenda 2063, the Maputo Plan of Action, and the Sustainable Development Goals.

We used a coding matrix for qualitative analysis to identify recurring themes of injustices and their underlying drivers. We used Arksey and O’Malley’s framework, guided by the Preferred Reporting Items for Systematic Reviews and Meta-Analyses Extension for Scoping Reviews (PRISMA-ScR) checklist, to inform research questions and literature identification, select papers, and chart, collate, and summarise the findings.

### Information sources

We sourced papers from PubMed, Scopus, Web of Science, Hein Online, Science Direct Online, and HINARI to ensure a comprehensive approach. We used a broad spectrum of sources, including authoritative scholarly opinions on SRH injustices. Grey literature papers were excluded because this review’s objective was to explore empirically reported evidence of SRHR injustices among adolescent girls. Focusing on peer-reviewed papers ensured methodological consistency and feasibility, considering the extensive grey literature in the SRHR field. Given that we conducted a scoping review of papers, we did not include primary sources of law, such as treaties or legislation.

### Search strategy

In this scoping review, the search strategy focused on papers that specifically integrated SRHR terminologies. We conducted comprehensive literature searches using search phrases to retrieve relevant resources. Using different terms and combining them with Boolean operators improved search accuracy (See additional table file 1). This method facilitated widening or narrowing the search in the identified databases. We standardised the search strategy in collaboration with the libraries at Utrecht University and Amref International University.

The search strategy required that papers included in the review must contain the keywords “Adolescent,” “sexual reproductive health,” and “Kenya,” “Zambia,” and “Ghana.” In HeinOnline, the legal database, keywords included “Constitution,” “Law,” “United Nations Treaties,” and “United Nations Committee Reports.” To broaden the search, search terms included the study population “adolescent girls in Kenya, Zambia, and Ghana aged 10–19” and the issue “sexual reproductive health,” as well as legal aspects such as “the constitution, Law, Treaties, and UN Committee reports.” Drivers were not included in the search terms to allow the research to identify the type of drivers the search would yield. The search was conducted on March 24, 2024 (See the attached Table 1 for the full search strings).

### Eligibility criteria

In line with JBI guidelines, we outlined inclusion criteria for the population, concept, and context as follows:

### Population

The population considered included adolescent girls aged 10 −19 years in Kenya, Ghana, and Zambia.

### Concept

We considered sexual and reproductive health and rights injustices to be violations of internationally agreed human rights law as set out in human rights conventions. These include gender-based violence, unsafe abortions, child/forced marriage, FGM/C, and lack of access to comprehensive sexuality education.

### Context

The context of the scoping review was in Kenya, Ghana and Zambia.

A comprehensive search framework was conducted for an extensive online search of relevant papers. The papers included were published between 2000 and 2024 to provide a broader scope of drivers based on emerging laws and ASRHR commitments by countries. This period marked major global and regional commitments to ASRHR, signaling the beginning of an era that shifted attention to rights-based and SRHR-focused programming in low- and middle-income countries. It also focuses on international, regional, and national policies. This 24-year period captures the interventions, providing a comprehensive understanding of progress, gaps, and persisting injustices. It is a period that brought about major global and regional commitments to ASRSR, marking the beginning of an era that shifted attention to rights-based and SRHR-focused programming in low- and middle-income countries. It also focuses on international, regional, and national policies.

We focused on adolescent girls aged 10–19 in Kenya, Ghana, and Zambia and considered papers that presented different groups but included girls aged 10–19 for full-text screening. We included papers addressing sexual and/or reproductive health and rights. We also included papers that presented an elaborate opinion on the drivers of SRH injustices to assess, analyse, evaluate, interpret, or critique them. We considered those that presented different groups but included girls aged 10–19 (e.g., girls aged 15–24 or both boys and girls) for full-text screening to confirm if they covered our specific age group. All the papers identified used qualitative, quantitative, and mixed methods.

### Selection process

A total of 648 records were identified through the comprehensive search strategy. Records from all databases were exported to Rayyan software for screening, except for HeinOnline, which was incompatible with the platform. As a result, records from HeinOnline were screened manually. Duplicate records were identified and removed using Rayyan for compatible databases and manually for HeinOnline, resulting in the removal of 191 duplicates and leaving 457 records for screening.

Two reviewers (D.A. and F.K.) independently screened titles and abstracts of the remaining 457 records. To ensure consistent application of the eligibility criteria, the reviewers jointly screened the first 30 records and resolved any discrepancies through discussion. The remaining records were screened independently, and discrepancies were resolved during this phase through discussion, with input from a third senior reviewer (R.G.) as needed. This stage resulted in the exclusion of 399 records that did not meet the inclusion criteria.

The remaining 58 full-text articles were then assessed for eligibility. Full-text screening was conducted independently by the same two reviewers. Disagreements were again resolved through discussion or with input from the senior reviewer (R.G.), where consensus could not be reached. Of the full texts assessed, 40 were excluded for not meeting the inclusion criteria, leaving 18 included in the scoping review.

Due to the methodological diversity of the included papers in this scoping review, the authors could not apply a single quality appraisal checklist. One author reviewed the papers narratively, assessing key methodological factors such as the appropriateness of the study design, objective clarity, and transparency in data collection, analysis, reporting of findings, and conclusions. The authors conducted this appraisal to contextualize evidence consistent with scoping review approaches. All the papers identified were peer-reviewed.

### Data synthesis

**Fig. 1:**
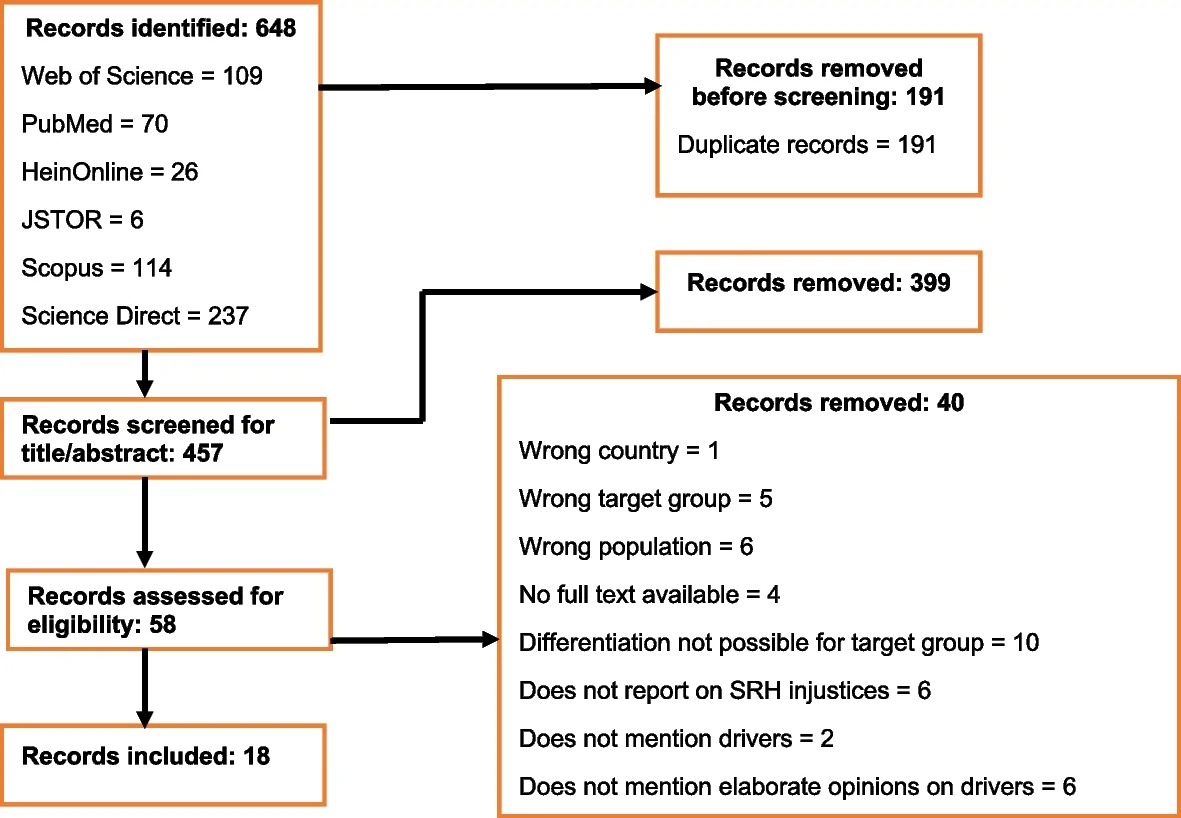
Identification of papers via databases

The authors used a scoping review and synthesized data from the identified papers. The process involved extracting drivers of SRHR injustices from each study. The data were reviewed across the different papers to identify recurring patterns and, iteratively, grouped comparable findings into thematic categories. The researchers refined the process by prioritizing issues that occurred frequently through repeated reviews of the charted data, ensuring they reflected the data extracted from the identified papers. This synthesis resulted in the final six themes, which represented the most significant drivers of adolescent SRHR injustices among the papers using the SRHR lens (Fig. 1).

This study included 18 [30–47] papers from the three countries after a rigorous screening. Additional table file 2 shows the characteristics of the 18 included papers. The data charted included the author’s last name, year of publication, aim of the paper, paper type, target population, age group, study location, driver, as is in the text, and type of injustice. We reviewed the selected papers and their drivers of injustices, categorising them into six key themes: poverty, culture and gender norms, the presence of an impairment, low levels of education, limited access to SRH services, and peer pressure. Thus, the study used these results to guide the narrative synthesis. Although some papers explicitly explored a single driver, others integrated more than one driver into their context.

### Characteristics and distribution of retrieved papers

Of the 18 papers included in this scoping review, 8 focused on different contexts within Ghana, 9 on Kenya, and 1 on Zambia, and within them, additional regional references to countries like Nigeria, Liberia, Zimbabwe, Congo, Malawi, and Uganda. The papers employed a variety of designs, reflecting the multidisciplinary nature of SRHR research. These included 2 scoping reviews, 1 systematic review, 3 quantitative cross-sectional studies, and a range of qualitative approaches, such as focus group discussions, in-depth interviews, ethnographic studies, and retrospective or comparative designs, for a total of 12. Additionally, one study employed a mixed-methods approach, and another used a longitudinal qualitative design. The target populations varied across studies and included adolescent girls and boys, youth with disabilities, children in the streets, refugees, deaf persons, and rape survivors, typically within the 10 to 19-year age range. Data charted on the aim of the paper, type of the paper, target population, age group, location, identified drivers of SRH injustices such as poverty, gender norms, disability, and legal or cultural barriers, and the types of injustices reported such as gender based violence, child marriage, limited access to SRH services, unsafe abortions, and transactional sex. These details are summarised in Additional table file 2, which provides a structured overview.

### Explanation and frequency of drivers

**Fig. 2:**
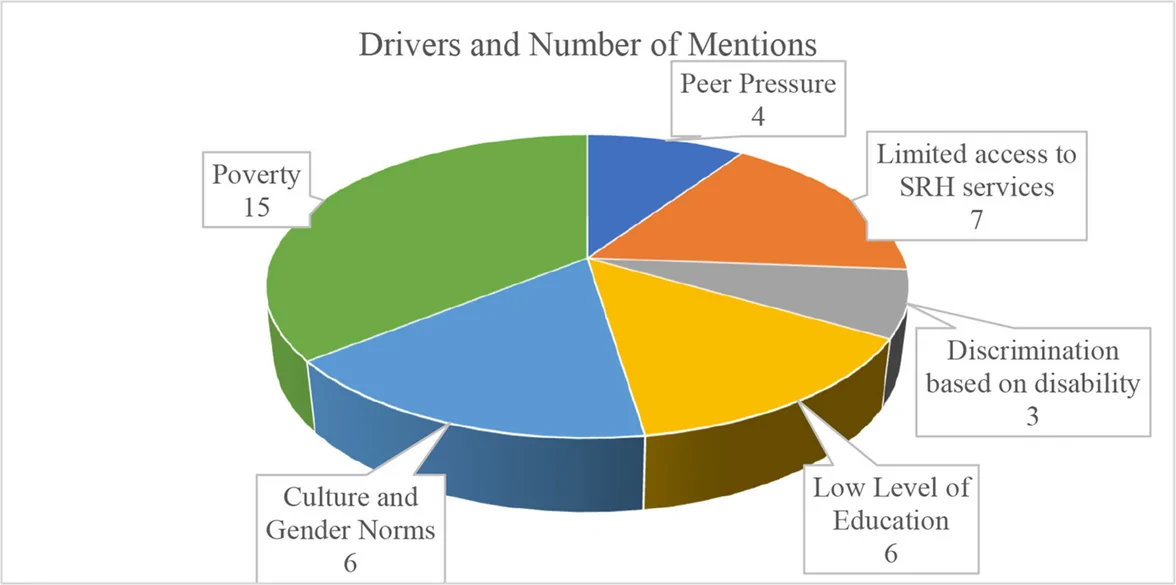
Frequency of mentions of drivers

A scoping review of the included papers generated six overarching themes that represent drivers of SRHR injustices among adolescent girls. To visualise the frequency with which specific ASRHR injustice drivers were mentioned in the articles, we mapped them as set out in Fig. 2 below. It reveals that the top three most common drivers of ASRHR injustices mentioned within papers explicitly using an SRHR lens are poverty, culture and gender norms, and limited access to SRH services. Poverty includes a range of diverse aspects, including a lack of resources to access SRH, a lower level of education, living in areas where there is limited access to SRH services, and being economically dependent. Dynamic cultural and gendered norms can drive violations of ASRHR, including, for example, the practice of female genital mutilation (FGM) and stigma around sex outside of marriage. Cultural practices linked to gender norms can also present barriers that limit girls’ knowledge of and access to SRH services, which was another frequent driver. While drivers can overlap in this way, these papers also established the uniqueness of each driver, given their complex yet subtle influences on the prevalence of injustices.

Limited access to SRH services was also identified as both an injustice and a driver, meaning that limited access leads to low knowledge of SRH and available services, which in turn makes adolescents less likely to seek and use these services. Other drivers, such as discrimination based on disability, low education level, and peer pressure, also overlap, even though they appear to be independent drivers in the papers. Our review noted that all the drivers are interrelated. With these connections, we also found sexual and reproductive health injustices associated with each driver. Each of these six identified drivers, along with the number of mentions in the reviewed papers (see Fig. 2), is addressed below.

#### Poverty

We identified poverty as the most often mentioned driver with widespread influence in varied ASRH injustices in the three countries. Based on the papers, we defined poverty as the compromised economic and lifestyle situation of adolescent girls, characterised by poor housing and lack of basic needs [34]. Some papers explicitly identify poverty as the central driver of sex work, sex for monetary support, and intergenerational transactional sex (ITS), which is when young people engage in sexual intercourse with older people in exchange for school fees, food, clothes, etc. [36, 38, 41]. Poverty had a direct impact on adolescent pregnancy [31–33, 35, 37, 39, 46], limited access to SRH services [37, 42, 43], and sexual abuse [41]. Our review uncovered that limited access to SRH services leads to adolescent pregnancies and concealed forms of sexual abuse.

#### Limited access to SRH services

Seven of our papers [31, 33, 35, 39, 42, 45, 48] identified limited access to SRH services as a driver of ASRH injustices in the three countries. For instance, the papers uncovered shortcomings such as poor access or unavailability of contraceptives, such as condoms, for this age group [31]. The emerging emphasis is that contraceptive use remains significantly low, causing unintended and early adolescent pregnancies. Factors such as access fees and inconvenient operating hours for healthcare facilities make it challenging for adolescents to access SRH services [35]. Healthcare professionals’ absence and long waiting times are also issues for this age group, driven by factors such as peer pressure [35, 39]. In remote rural areas, services are entirely scarce or unavailable [39]. Such situations make it difficult for sexually abused adolescents to receive comprehensive examinations and treatment or to procure prosecutorial evidence for use in courts [45]. The quick dismissal of cases due to a lack of services exposes adolescents to further sexual abuse risks [45]. Other papers indicate that denying adolescents below the age of consent SRH services further exposes them to unprotected sexual encounters and pregnancies [33, 42].

#### Culture and gender norms

##### Harmful cultural practices

Culture and gender norms were the third most often mentioned common drivers of ASRH injustices. The papers revealed that cultural practices, such as patriarchy, which include the beliefs and norms that privilege/position men over women and bestow them with control over certain aspects, such as bride price (dowry), marriage, and sexual intercourse, leading to early pregnancies [31]. In some instances, the bride price for adolescent young girls increases their vulnerability to gender-based violence due to accompanying gender values and societal obligations, such as early marriages [31, 36, 37]. We found that bride price reinforces harmful gender practices that lead to perceived ownership/domination and coercion of the adolescent bride by the groom, leading to girls’ silence while in abusive relationships [31, 36]. Some of the papers explicitly underlined marriage and childbirth as a fundamental duty for girls in Ghana [36], which can expose them to adolescent pregnancy and sexual abuse [32]. There are cultures where adolescent marriage is the acceptable norm, and young girls are expected to endure such practices, similar to their foremothers [40].

The papers extensively flagged gender and cultural norms as a major limiting factor for adolescents’ access to SRH services. For instance, they report that the recognition of sexually related information as taboo [39] in some of the Ghanaian and Kenyan contexts leads to withholding SRH information. We found that taboos and culture are closely correlated and hamper teenagers’ ability to access SRH services because they cannot speak about sex-related issues. Papers indicate that culture limits parent-daughter sex education [39] and that fathers rarely provide any sex-related guidance to their daughters [35]. Information sharing is a significant issue that predisposes adolescent girls to early pregnancy, sexual abuse, and intergenerational sex [35, 39]. The desire to have many children for social status and reputation is a cultural issue that limits demand for SRH services, with some perspectives linking women using contraceptives with infidelity and promiscuity [36].

Some cultural norms also prevent girls from receiving SRH services from male workers, limiting access to such services. The only available information is the traditional teachings [35] and their timeline coincides with marriage. We found that the community’s expectations for girls to meet their childbearing responsibilities create barriers to access SRH services because they prefer traditional methods rather than going for specialised hospital care [36]. Further, other papers indicate that cultural factors, such as traditional African beliefs, influence drivers of injustice [32, 35, 36]. The findings reveal that culture affects access to SRH services by limiting information for adolescents and, in other cases, identifying some practices as taboos [32].

##### Protective cultural practices

Results indicate that some cultural practices and gender norms can also function to prevent SRHR violations. One of the papers acknowledged that the cultural prohibition of premarital sex encourages adolescents to abstain until married [34], which can limit the spread of sexually transmitted diseases and unwanted pregnancies. Others indicate that gender norms requiring girls to be in the home performing chores can function in effect to protect them from sexual abuse risks, early sex debut, and pregnancies [34]. Although other cultural practices, such as virginity inspection and puberty rites, are declining in the region [39], they can also function to delay sexual activity and some SRHR injustices (despite potentially also being a human rights violation themselves [48]).

#### Low educational levels

Low education levels were mentioned as a leading driver for specific ASRH injustices, including adolescent pregnancy and inadequate access to SRH services. Researchers reported that a poor understanding of menstrual cycles and a lack of awareness about contraceptives and their uses contribute to persistent adolescent pregnancies in the three countries [31]. Within the school setting, teachers often provide adolescents with limited SRH information, thereby maintaining their low educational levels [35]. Other papers revealed that poor reproductive health knowledge or unawareness of subtle sexuality issues is a common problem among out-of-school adolescents [39]. Some young girls in countries such as Ghana remain incapable of making decisions about whether to engage in intimate relations or whether to use protection despite reaching the legal age of consent, which is 16, due to their low levels of education. Girls are sometimes coerced into having sex, leading to unprotected sexual encounters and teenage pregnancies, not to mention sexual assault [35]. Research also confirms that due to cultural/gender norms or early pregnancy, girls who marry early drop out of school, which denies them education and further undermines their ability to know about or access SRH services [37, 44].

#### Peer pressure

Our scoping review identified peer pressure as a direct and indirect driver of adolescent pregnancy. For instance, one of the papers examining the popularisation of intergenerational transactional sex among Kenyan adolescents uncovered that the pressure to live “a better life” was highest among adolescent mothers and school dropout adolescents [41]. Due to this pressure, some adolescent girls succumb to transactional sex, where they have minimal control over the use of contraceptives (condoms), exposing them to unplanned pregnancy. As noted above, poverty closely relates to sex work as adolescents exchange sex for food and money [48]. A significant proportion of peer pressure also occurs through social media, which causes adolescent curiosity and the desire to fit in [39]. The influence also includes the normalisation of multiple sexual partnerships as a survival tactic and a longing for social grouping recognition [34, 47]. This peer pressure can perpetuate unhealthy sexual practices and lead to increasing SRHR injustices.

#### Discrimination based on disability

Lastly, we identified having disability related exclusion and discrimination as a driver for SRHR injustices like adolescent pregnancy. One paper indicated that the inability to access appropriate contraceptives or their awareness among deaf girls exposes them to unprotected sex, leading to unwanted pregnancies [37]. The lack of specialised personnel to communicate with them complicates government efforts to provide fundamental SRH services in Ghana, resulting in inadequate or unavailable services [37]. Findings also indicate that persons with a disability, particularly those with intellectual disability, face challenges accessing similar services due to communication issues [43].

### Drivers of a common injustice: adolescent pregnancy

**Fig. 3:**
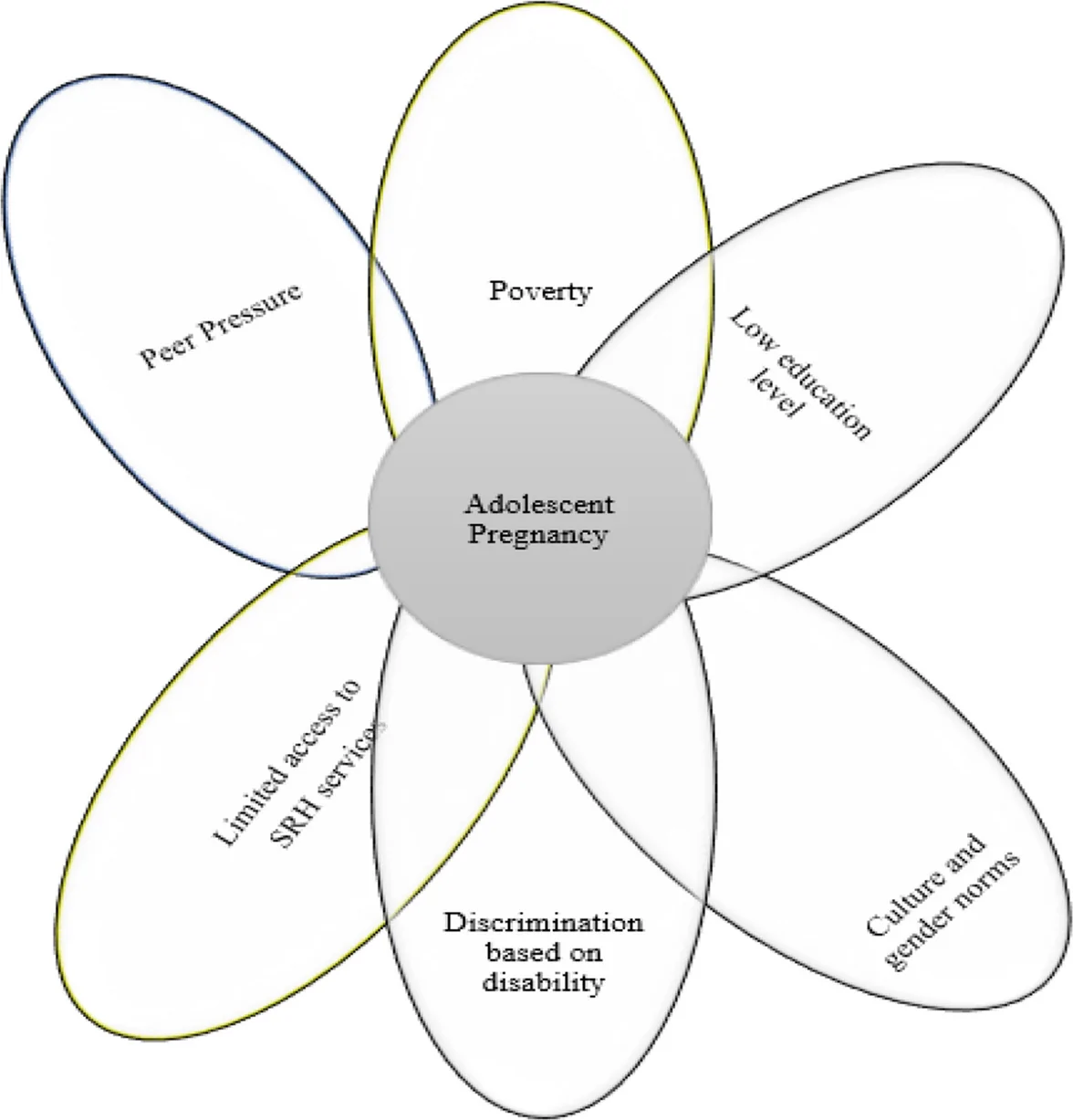
Drivers’ interrelationships and the emerging common ASRH injustice

Our review found that all the drivers contribute to the occurrence of adolescent pregnancy as a common SRHR injustice. Lack of awareness about appropriate SRH care due to low education complicates access to SRH services, exposing adolescents to unplanned pregnancies and sexual abuse [36]. The effects of culture in the three countries limit the provision of SRH information in schools, leading to low education levels on sexuality, therefore increasing vulnerability to sexual abuse and early pregnancy [35]. Further, low education levels arising from cultural beliefs discriminate against girls accessing education and encourage early marriages, which is also an injustice [36]. Other papers indicate that adolescent pregnancy and other injustices occur because of poverty, making it unaffordable to pay access fees for SRH goods, facilities, and services [35, 37]. Poverty, together with peer pressure, exposes adolescent girls to intergenerational transaction sex, among others, which can result in unwanted early pregnancies [41]. Therefore, such intricate interconnections underscore the complex inter-relationships between the drivers and ASRHR injustices, as depicted in Fig. 3 below.

## Discussion

We found that poverty, culture and gender norms, limited access to SRH goods, facilities and services, low education levels, peer pressure, and the presence of discrimination based on disability were the six leading drivers of adolescent SRHR injustices in the selected papers for girls in Kenya, Ghana, and Zambia. The injustices raised in the papers include FGM/C, sexual violence, gender-based violence, child marriage, lack of services, and lack of comprehensive sex education. Limited access to SRH services stands out as both a driver of injustices and an injustice itself.

Of the varying types of injustices, our review found that each of the six drivers influenced the rate of adolescent pregnancy. Adolescent pregnancy itself is not a human rights violation. It often relates to violations such as child marriage, lack of access to SRH information and services (including contraceptives and abortion), forced pregnancy, or (statutory) rape. In the same way, getting a sexually transmitted disease is not a human rights violation, but a lack of access to SRH services is [49]. Similarly, some of the drivers are themselves not a human rights violation (i.e., having an impairment), but rather can lead to human rights violations (discrimination).

Cultural and gender norms are also not human rights violations in themselves, except where they promote or perpetuate discrimination and gender inequality, according to the CEDAW [5]. While poverty per se is not a human rights violation, under the ICESCR [11], the lack of access to essential goods, such as food, water, shelter, or clothing, constitutes a human rights violation. This relates to the fact that human rights are all interdependent, interrelated, and indivisible [50]. Human rights reflect the complex matrix of human lives and, therefore, require holistic, not piecemeal, approaches to their protection.

Some of these papers are not nuanced enough. Therefore, the drivers base their intersectional analysis on a layered, interrelated approach [51]. These present double injustices among adolescent girls that perpetuate gender inequality and discrimination. It limits their access to education and healthcare, thus providing us with an understanding of how these vulnerabilities are layered and interrelated. The papers investigating the interactions among drivers, including culture and gender norms, poverty, and education levels, uncovered the interrelationships between drivers and how they worsen each other. The results bring out the intersection of numerous forms of discrimination among adolescent girls.

Scholarship has developed and elaborated on intersectionality, examining how different characteristics intersect and manifest in discriminatory practices [52, 53]. In our scoping review, relevant grounds of intersecting discrimination include age, sex, class, poverty, and race. For example, all of these characteristics can be relevant in intergenerational transactional sex, particularly where the older man is of a different race, which is not uncommon. While it certainly occurs in the three countries studied, our review did not reveal trafficking as a driver or SRHR injustice, despite it being a driver and an injustice. Trafficking has been acknowledged as a socio-economic injustice requiring the adoption of transformative approaches to dismantle the structural and systemic conditions that deprive adolescent girls of their SRHR [54–56].

While the review identified various drivers of adolescent sexual reproductive health injustices, it did not highlight the increasingly prevalent impact of climate change in these three countries [57–59]. Our review identified limited evidence directly linking climate changes and ASRHR in papers that explicitly focus on SRHR. However, this outcome is not necessarily an indicator of the lack of literature on climate change and SRHR, but perhaps a suggestion that such papers have not framed these issues within the SRHR lens. Specific papers have demonstrated that climate change can lead to economic instability through droughts or floods that wipe out a family’s earning capacity, such as crop production, and due to cultural and gender norms or practices, this economic instability may result in increasing girls’ early marriages to bring in a bride price [60].

Extreme weather events like floods can also disrupt access to SRH services, impacting the rights of women and girls, particularly in marginalised communities in developing countries, including Kenya, Zambia, and Ghana [59]. Therefore, addressing these layers of vulnerabilities requires a comprehensive approach that integrates [61]. SRHR into climate change adaptation and building resilience, such as ensuring access to SRHR services and adopting gender-responsive climate action. Given that the impacts of climate change are expected to intensify, we suggest that this topic warrants further study [62].

### Strengths and limitations

While many scoping reviews have explored ASRH injustices, we believe that this is the first study targeting papers using an SRHR lens to investigate the drivers of these injustices among adolescent girls in Kenya, Zambia, and Ghana. We found that most papers focus on identifying ASRH injustices with minimal exploration of the dynamic mechanisms that cause these issues among the girls in communities. Although some reviewed papers directly link these injustices to specific drivers [31, 36, 41], others can only provide indirect associations because their methodologies do not allow for causal inferences.

The scoping review uncovered inter-relationships among drivers that require additional research to understand how they influence the persistent injustices despite preventive legal frameworks in the three countries. The results of our study revealed a dearth of research within the SRHR discourse examining the various drivers of SRHR injustices affecting adolescent girls, resulting in a limited number of papers (18) identified in this scoping review. However, we acknowledge that our intention to restrict our search to papers directly framed using the SRHR terminologies excluded study did not include all papers examining various drivers of ASRH injustices without necessarily framing them as SRHR issues. Our findings, therefore, reflect literature within SRHR framed papers and not totality of all SRHR issues affecting adolescents in the study regions. Another limitation was the lack of papers from Zambia, suggesting that there may be unique challenges or vulnerabilities that are not fully captured in the existing findings. A challenge that arose during the search was the inability to systematically search the African Journals Online (AJOL) database, which contains African-only papers that could have provided valuable information.

## Conclusion

This review examined the drivers leading to adolescent SRHR injustices in Kenya, Ghana, and Zambia in papers using an ASRHR lens. Poverty stands out and is interconnected with other factors, including a lack of education, access to SRH services, and cultural aspects. All of the drivers were related to adolescent pregnancy. While adolescent pregnancy in itself may not be a human rights violation, it may relate to violations such as lack of access to services, school dropout, lack of education, and child marriage. This comprehensive mapping of key drivers of ASRHR injustices should inform the (re)design, implementation, and evaluation of laws and policies that address adolescent girls’ vulnerability to SRH injustices. It may guide the programming of local civil society interventions focusing on ASRH. This mapping will inform reforms to the comprehensive sexuality education curricula that are age-appropriate across primary and secondary schools. Further, improvements to policies and laws will provide explicit legal protections for adolescents’ access to SRH services, as enforcement remains weak. We noted that some areas have received limited scholarly attention and thus require further study, such as disability among adolescent girls, as well as the increasing impact of climate change on sexual and reproductive health.

## Supplementary Information

Supplementary Material 1.

Supplementary Material 2. [31–48].

Supplementary Material 3.

## Data Availability

Not applicable.

## Abbreviations

AJOL: African Journals Online
ASRHR: Adolescent Girls’ Sexual Reproductive Health Rights
CEDAW: Convention on the Elimination of All Forms of Discrimination Against Women
FGM: Female Genital Mutilation
ICESCR: International Covenant on Economic, Social, and Cultural Rights
ICPD: International Conference on Population and Development
ITS: Intergenerational Transactional Sex
MDG: Millennium Development Goals
PRISMA-ScR: Preferred Reporting Items for Systematic Reviews and Meta-Analyses Extension for Scoping Reviews
SDG: Sustainable Development Goal
SRH: Sexual Reproductive Health
SRHR: Sexual Reproductive Health Rights
SSA: Sub-Saharan Africa
UNDP: United Nations Development Programme
UNFPA: United Nations Population Fund
UNICEF: United Nations Children’s Fund
