# Supplementary material for: Mapping drivers of sexual and reproductive health injustices among adolescent girls in Kenya, Ghana, and Zambia: a scoping review
Source: BMC Public Health. 2026 Aug 11;26:3239. doi: 10.1186/s12889-026-28865-1 (PMC13741468; doi:10.1186/s12889-026-28865-1)
Supplement: Supplementary file 1 [file 12889_2026_28865_MOESM1_ESM.docx]

**Additional File 1**

| **Database** | **Full search string – with Boolean** | **Filters Applied** | **Results** |
| --- | --- | --- | --- |
| PubMed | ((((Adolescen* [Title/Abstract] OR Youth* [Title/Abstract] OR young* [Title/Abstract] OR Teenage*[Title/Abstract]) AND (“reproductive health” [Title/Abstract] OR “sexual health” [Title/Abstract] OR “reproductive well-being”[Title/Abstract] OR “sexual well-being”[Title/Abstract)) AND right* [Title/Abstract]) AND (Kenya* OR Zambia* OR Ghana*[Title/Abstract])) | Publication date from 2000 - 2024  Text availability – Title/Abstract  English | 70 |
| Web of Science | ((((Adolescen* OR Youth* OR young* OR Teenage*) AND (“reproductive health” OR “sexual health” OR “reproductive well-being” OR “sexual well-being”)) AND right*) AND (Kenya* OR Zambia* OR Ghana*)) | Publication date from 2000– 2024  Text availability - Topic  English | 109 |
| Scopus | (adolescen* OR youth* OR young* OR teenage*) AND (“reproductive health” OR “sexual health” OR “reproductive well-being” OR “sexual well-being”) AND right* AND (Kenya* OR Zambia* OR Ghana*) | Publication date from 2000 – 2024  Text Availability- Title, Abstract, Keywords  English | 114 |
| HeinOnline | ((((((Adolescen* OR Youth* OR young* OR Teenage*) AND (Kenya* OR Zambia* OR Ghana*)) AND (right*)) AND (Constitution* OR Law* OR “United Nations Treaties” OR “United Nations Committee reports”)) AND title:( “reproductive health” OR “sexual health” OR “reproductive well-being” OR “sexual well-being” ))) | Publication date from 2000 – 2024  Text Availability- International, human rights and african law journals  all are full text, except for SRH string: title | 26 |
| SCIENCE DIRECT | adolescence OR adolescents AND Kenya OR Ghana OR Zambia AND “sexual health” OR “reproductive health” or “Sexual well-being” OR “reproductive well-being” AND rights | Publication date from 2000-2024  Title/abstract/keywords  English | 237 |
| JSTOR | ((((ab: “Kenya* OR ghana* OR Zambia*”) AND (ab: “adolescen* OR young* OR youth* OR teenage*”)) AND (ab: ““reproductive health” OR “sexual health” OR “reproductive well-being” OR “sexual well-being”)) AND (ab: “right*”)) | Abstract  2000-2024  English | 6 |
| HINARI | (Abstract:( “sexual health” OR “sexual well-being” OR “sexual well being” OR “reproductive health” OR “reproductive well-being” OR “reproductive well being”)) AND (Abstract:(adolescen* OR youth* OR young* OR teenage*)) AND (Abstract:(right*)) AND (Abstract:(Kenya* OR Ghana* OR Zambia*)) | Abstract  2000-2024  Journals | 86 |
