# Supplementary material for: Mapping drivers of sexual and reproductive health injustices among adolescent girls in Kenya, Ghana, and Zambia: a scoping review
Source: BMC Public Health. 2026 Aug 11;26:3239. doi: 10.1186/s12889-026-28865-1 (PMC13741468; doi:10.1186/s12889-026-28865-1)
Supplement: Supplementary file 2 [file 12889_2026_28865_MOESM2_ESM.docx]

**Additional Table 2: Charting Table for Reviewed Papers**

| **No** | **Author’s Last Name** | **Year of publication** | **Aim of the paper** | **Paper type** | **Target population** | **Age group** | **Study location** | **Driver as is in the text** | **Type of injustice** |
| --- | --- | --- | --- | --- | --- | --- | --- | --- | --- |
| 1 [31] | Wado et. al. | 2020 | To map and describe available research and evidence on adolescent SRHR in slums in SSA | Scoping review | Adolescent boys and girls who live in slum areas | 10-19 | Kenya | - Urban poverty  -traditional norms and cultural beliefs  -low educational attainment | Gender-based violence, lack of access to SRH services, adolescent pregnancy |
| 2 [32] | Lambonmung et al. | 2022 | To Highlight the health impacts of adolescent pregnancy through a systematic review | Systematic review | Adolescent girls | 10-19 | Ghana, Nigeria, Liberia | -Poverty | Gender-based violence, inequities, unsafe abortions |
| 3 [33] | Chandra-Mouli et al. | 2019 | To analyse epidemiological trends of factors that helped or hindered progress in achieving comprehensive sexuality education around adolescent pregnancy and childbearing | Retrospective qualitative | Adolescent girls | 10-19 | Global (mentioning Kenya and Ghana) | -Persistent poverty  -child marriage (culture)    -legal barriers  to access to SRH services | Gender-based violence, adolescent pregnancy, child marriage, FGM, lack of access to comprehensive sexuality education |
| 4 [34] | Omanga et al. | 2023 | To assess three sexual risk behaviours (sexual debut, multiple sexual partners and intergenerational sex) among youth and also understanding local contexts of these practices to inform program design | A cross-sectional formative qualitative study | Male and female youth, parents, teachers, community leaders, district leaders and religious leaders | 15-17 | Kenya | -poverty and limited unemployment opportunities  -peer pressure  for early sexual debut | Intergenerational sex among children |
| 5 [35] | Langat et al. | 2024 | To understand the challenges and opportunities for improving access to SRH services | Qualitative study | Adolescents and youth both male and female | 15-19 | Kenya | -gender, culture, and  religion influenced AY access  -low or complete lack of SRH  education  -lack of SRH service awareness | Lack of comprehensive sexuality education |
| 6 [36] | Markwei et al. | 2022 | Explore the SRH experiences of victims of child marriages, examine how the social position of girls influences their experiences in marriage | Qualitative design (semi-structured interviews) | Adolescent girls and young women who were married before the age of 18^22^ | 14-21 | Ghana | Economic difficulty  Power structure and patriarchal norms | Child marriage, adolescent pregnancy, not having access to pre-and postnatal health care |
| 7 [37] | Nketsia et al. | 2022 | Develop an in-depth understanding of the knowledge of deaf persons regarding services such as knowledge of contraceptive methods, pregnancy and safe abortion practices | Sequential explanatory mixed-methods approach | Deaf persons | 17-46 | Ghana | -Having an impairment  -Low education  -Poverty  -Limited avenues to receiving SRH information | Lack of access to SRH services |
| 8 [38] | Morhe et al. | 2019 | To examine the social and ethical challenges in enforcing sexual and reproductive rights of male and female adolescents abused at sexual debut in Ghana | Cross-sectional quantitative survey | Sexually inexperienced male and female teenagers | 10-19 (girls were 12-19) | Ghana | -Low educational among deaf people and need for financial support | Sexual abuse, rape, early sexual debut, adolescent pregnancy |
| 9 [39] | Amoadu et al. | 2022 | Summarise studies published on socio-cultural determinants of adolescent pregnancy in Ghana | Scoping review | Adolescents | 10-19 | Ghana | -Transactional sex for money (Poverty)  -peer influence  -low level of education  -child marriage,  coerced sex (culture)  -non-usage of contraceptives,  and decline in cultural values in Ghana | Adolescent pregnancy |
| 10 [40] | Ako et al. | 2009 | Summarises the ineffectiveness and challenges of the law in curbing FGM | Qualitative survey | Adolescent boys and girls, young men and women and adults | 15-24 | Ghana | -FGM is  embedded in the culture | FGM |
| 11 [41] | Zamudio-Haas et al. | 2021 | Provide insights into the structural inequities and poverty that motivate their peers to engage in intergenerational transactional sex relationships with older men and women | Focus group discussions (qualitative) | Adolescent boys and girls | 15-19 | Kenya | -Poverty is a driver of sponsored relationships  -Inability to  remain celibate due to peer and other pressures | Intergenerational transactional sex, adolescent pregnancy |
| 12 [42] | Kangaude et al. | 2020 | Focuses on age and gender to help shed light on whether there is progress towards UHC and other global commitments for adolescent SRH | Comparative study design using legal analysis and in-depth interviews (Qualitative) | Adolescents | 10-19 | Zambia including Malawi and Ethiopia | -Economic issues | Unsafe abortions |
| 13 [43] | Tanabe et al. | 2015 | Explore risks, needs, and barriers for refugees with disabilities to access SRH services, and the practical ways in which these challenges could be addressed | Qualitative study design | Refugee men and women with and without impairment | 15-19 | Kenya, Nepal, Uganda | -Lack of awareness, limited access to information, especially those with intellectual impairments | Access to SRH services, sexual violence, forced abortion, forced use of contraception, forced sterilisation |
| 14 [44] | Kumi-Kyereme et al. | 2020 | Challenges young people with disabilities faced in accessing SRH services | Quantitative cross-sectional study | Adolescent and youth with disabilities | 10-19 and 20-24 | Ghana | -Education level showed challenges in accessing SRHS  -Poverty  -PLWDs | Inaccessibility of SRH services |
| 15 [45] | Wangamati et al. | 2016 | Highlight SRH and rights observations and violations experienced by minor seeking post-rape care services | Qualitative study | adolescent girls, rape victims | 14 | Kenya | -Socio-cultural traditions, cultural perceptions  -Inaccessibility of services | Access to SRH services (for rape) |
| 16 [46] | Karp et al. | 2021 | Understand how COVID-19 restrictions affected girls’ and young women’s relationship experiences, examine drivers of these changes and explore how these changes inform youth SRH and well-being | Mixed methods (surveys) | Girls and young women | 15-24 | Kenya | -Economic hardships  -Poverty and loss of financial resources | Sexual violence, early pregnancy, transactional relationships |
| 17 [47] | Hunter et al. | 2021 | Explore the interaction between peer relationships and sexual health among street youth in three SSA countries | Longitudinal research project (qualitative) | Street youth | 14-20 | Ghana, Zimbabwe, Congo | -Poverty  -Peer pressure | Unsafe abortions, receiving misinformation about contraceptives, access to SRH services |
| 18 [48] | Embleton et al. | 2015 | Explore the sexual language and practices of street-connected children and youth in western Kenya | Qualitative research (in-depth interviews) | Girls and boys | 11-24 | Kenya | Poverty and challenges in implementing and scaling up adolescent-specific programmes  Lack of access to SRH services^34^ | Sexual relations below the age of consent (16 years in Kenya), the SGBV endured by girls and exploitation while living in the streets and transactional sex |
